# The functional impact on donor vessel following transcatheter closure of coronary artery fistulas—a retrospective study using QFR analysis

**DOI:** 10.3389/fcvm.2024.1435025

**Published:** 2024-07-31

**Authors:** Zhenchi Sang, Qingqi Ji, Huan Tong, Linghong Shen, Xiaolong Wang, Ben He

**Affiliations:** ^1^ Department of Cardiology, Shanghai Chest Hospital, Shanghai Jiao Tong University School of Medicine, Shanghai, China; ^2^ Department of Cardiology, Shuguang Hospital, Shanghai University of Traditional Chinese Medicine, Shanghai, China

**Keywords:** coronary artery fistula, transcatheter closure, donor vessel, QFR, residual shunt

## Abstract

**Background:**

The impact of transcatheter closure of coronary artery fistula (CAF) and residual shunt after occlusion on improving blood flow in the donor vessel remains uncertain.

**Objectives:**

To evaluate the functional impact on the donor vessel following CAFs closure using QFR (Quantitative Flow Ratio) analysis.

**Methods:**

A total of 46 patients with 48 CAFs who underwent transcatheter closure at Shanghai Chest Hospital and Shuguang Hospital between March 2015 and August 2023 were included in the review. The clinical, angiographic details, and QFR data were subjected to analysis. The size of the fistulae was defined according to the ratio between the diameters of the fistulae and the largest diameter of the coronary vessel not feeding the coronary fistula.

**Results:**

Among 48 CAFs, the average diameter of the fistulae ostium was 3.19 ± 1.04 mm, while the mean diameter of the donor vessel segment following fistulae was 3.45 ± 1.01 mm. The mean QFR value of the donor vessels with medium CAFs was found to be significantly lower than those with small CAFs (0.93 ± 0.10 vs. 0.98 ± 0.03; *p* < 0.05). Furthermore, the mean QFR value of donor vessels with medium CAFs was observed to be significantly improved after occlusion (0.99 ± 0.01 vs. 0.93 ± 0.10; *p* = 0.01). However, there was no statistical difference in the mean QFR value of donor vessels with small CAFs before and after occlusion (0.98 ± 0.03 vs. 0.98 ± 0.02; *p* > 0.05). Moreover, the changes in QFR were more pronounced in donor vessels with medium CAFs compared to those with small CAFs after occlusion (0.06 ± 0.10 vs. 0.005 ± 0.012; *p* = 0.01). There is no statistical difference in the mean QFR variation and QFR variation rate between donor vessels with CAFs that occurred residual shunt and those without residual shunt after occlusion (*p* > 0.05).

**Conclusions:**

The presence of medium CAFs has a significant impact on the blood flow of the donor vessel, as compared to small CAFs, and may benefit from occlusion. A small residual shunt has no significant impact on the effectiveness of CAFs occlusion in enhancing donor blood flow.

## Introduction

Coronary artery fistulas (CAFs) are rare anomalies of the coronary arteries, typically communicating with the cardiac chambers, venous circuits, or pulmonary circulation. The population incidence rate is 0.1% (1, 2), and small CAFs are generally asymptomatic, usually diagnosed incidentally during coronary angiography or non-invasive coronary imaging (3). Medium or large CAFs may result in myocardial ischemia, heart failure, arrhythmia, and infective endocarditis (4). The presence of CAFs with elevated shunt flow is highly suggestive of a high probability of coronary artery steal.

Currently, the principal therapeutic modalities for medium- or large-sized CAFs are surgical ligation and percutaneous transcatheter closure (5). The interventional devices utilized for the transcatheter closure of CAFs are predominantly coils and vascular occluders (6).

The current evaluation of the effect of transcatheter closure of CAFs mainly focuses on fistula blood flow and residual shunt, while the functional improvement of blood flow in the donor vessel is lacking. Nevertheless, the impact of total occlusion or residual shunt of CAFs on increased blood flow in the donor vessel remains elusive. A non-invasive method to quantify the physiological effect of the fistula may be a valuable addition to the existing toolkit.

This study aims to assess the impact of CAFs occlusion and residual shunt on blood flow in donor vessels using QFR.

## Materials and methods

### Patients

This is a retrospective study involving 46 patients with 57 CAFs presenting symptoms of myocardial ischemia, heart failure, or arrhythmia who were admitted to Shanghai Chest Hospital affiliated to Shanghai Jiao Tong University School of Medicine and Shuguang Hospital affiliated to Shanghai University of Traditional Chinese Medicine between March 2015 and August 2023. Patients were identified through a review of the catheter lab database. A comprehensive set of clinical, imaging, laboratory, cardiac catheterization, and procedural details was collected.

Patients with additional complex cardiac defects requiring surgical treatment, donor vessels with CAFs exhibiting significant atherosclerotic stenosis, excessively twisted donor vessels, or CAFs that could not be evaluated using QFR were excluded. Written informed consent was obtained from all patients or their legal guardians prior to cardiac catheterization and angiography.

### Devices

The occlusion devices utilized to close CAFs were included: Amplatzer vascular plug (AGA Medical Corporation, Golden Valley, MN); and Cook coil (Cook Cardiology, Bloomington, IN). The cook coils were delivered by a microcatheter, and the plugs were deployed by using a guiding catheter. Device selection was based on the specific anatomic characteristics of the fistula.

### Procedure

Following the provision of informed consent, dual antiplatelet therapy was preloaded in all patients. The procedures were conducted via radial or femoral arterial access, and unfractionated heparin was administered (100 IU/kg). Selective coronary angiography was performed to illustrate the anatomy of the fistula, encompassing its origin, termination site, and size. CAFs can be categorized as small, medium, or large based on the diameter of the fistula, which is defined as being <1, 1–2, or >2 times the largest diameter of the coronary vessel not feeding the coronary fistula, respectively (7). Antegrade deployment was preferred in all patients. The successful procedure is defined as the total occlusion of the coronary artery fistula following transcatheter closure, or the presence of a residual shunt with a significant reduction in blood flow.

### QFR measurement

QFR (Quantitative Flow Ratio) is computed on a Windows-based computer (AngioPlus Core v2.0; Shanghai Pulse Medical Technology, Inc.). Properly positioned angiographic runs are transferred to the QFR workstation and analysis is performed for all study donor vessels. QFR analysis is performed in accordance with the study-specific standard operating procedure as previously described in detail (8). The diameter of the fistula ostium and donor vessel after fistulae origin was quantified by QCA (Quantitative Coronary Angiography). A single end-diastolic frame with an optimal position is selected, and the segmented vessel contours are traced. The contrast frame count was performed during an angiographic run in which contrast movement was clearly visible.

The variations in QFR and IMR (Index of Microcirculatory Resistance) were defined as the difference between their respective values post-occlusion and pre-occlusion. The variation rates of QFR and IMR were defined as the proportion of their respective changes relative to the pre-occlusion value.

### Data analysis

The Institutional Research and Ethics Board approved the study. Clinical, angiographic details, and QFR data were analyzed. All angiographic images of patients were measured by the QFR system. Categorical variables are expressed as percentages and continuous variables are presented as mean ± standard deviation (SD). Differences in parameters before and after the procedure were compared using a paired *t*-test. Continuous variables were compared between two groups using the *t*-test. A *p* < 0.05 value was considered a statistical difference and a *p* < 0.01 value was considered a statistically significant difference. Statistical analysis was performed using SPSS version 21.0 (IBM Corporation).

## Results

A total of 46 patients with 57 CAFs, presenting symptoms of myocardial ischemia, heart failure, or arrhythmia, were admitted to Shanghai Chest Hospital affiliated to Shanghai Jiao Tong University School of Medicine, and Shuguang Hospital affiliated to Shanghai University of Traditional Chinese Medicine, between March 2015 and August 2023. The average age of 46 patients was 63.14 ± 9.00, with 23 patients being male (50%). Among them, 48 CAFs that underwent successful occlusion were deemed suitable for QFR analysis. The majority of patients (*n* = 35; 76%) exhibited a single fistula, while 11 patients had two fistulas originating from both the left and right coronary systems (24%). Devices were deployed using Coil by transcatheter in 47 CAFs (98%), while the remaining one CAF was treated with an Amplatzer vascular plug. On average, 47 CAFs were closed by using 3 coils. The mean diameter of the fistulae ostium was 3.19 ± 1.04 mm; correspondingly, the mean diameter of the donor vessel segment after fistulae was 3.45 ± 1.01 mm. Of the 48 fistulas, 22 were classified as medium, representing 46% of the total. The majority of the fistulas originated from the left anterior descending artery (LAD) (42%) and the right coronary artery (RCA) (50%). Only 2 fistulas originated from the left circumflex (LCX) and left main (LM), respectively. The most frequent drainage sites were the pulmonary artery (*n* = 26; 54%), right ventricle (*n* = 2; 4%), right atrium (*n* = 4; 8%), and left atrium (*n* = 16; 33%). The details of CAFs are listed in Table 1.

**Table 1:** Characteristics of coronary artery fistulas.

CAFs	*N* = 48
Size of the fistula	
Small	26 (54%)
Medium	22 (46%)
Large	0
Fistula origin	
LAD	20 (42%)
LCX	2 (4%)
RCA	24 (50%)
LM	2 (4%)
Fistula drainage	
PA	26 (54%)
RV	2 (4%)
RA	4 (8%)
LA	16 (33%)
Fistula diameter (mm)	3.19 ± 1.04
Donor vessel diameter (mm)	3.45 ± 1.01
Device used to occlude fistulae	
Coil	47
Amplatzer vascular plug	1
Procedural outcome	
Residual shunt	22
No residual shunt	26
Fistula diameter/donor vessel diameter	0.98 ± 0.33

CAF, coronary artery fistula; LAD, left anterior descending; LCX, left circumflex; RCA, right coronary artery; LM, left main; PA, pulmonary artery; RV, right ventricle; RA, right atrium; LA, left atrium.

**Figure 1:**
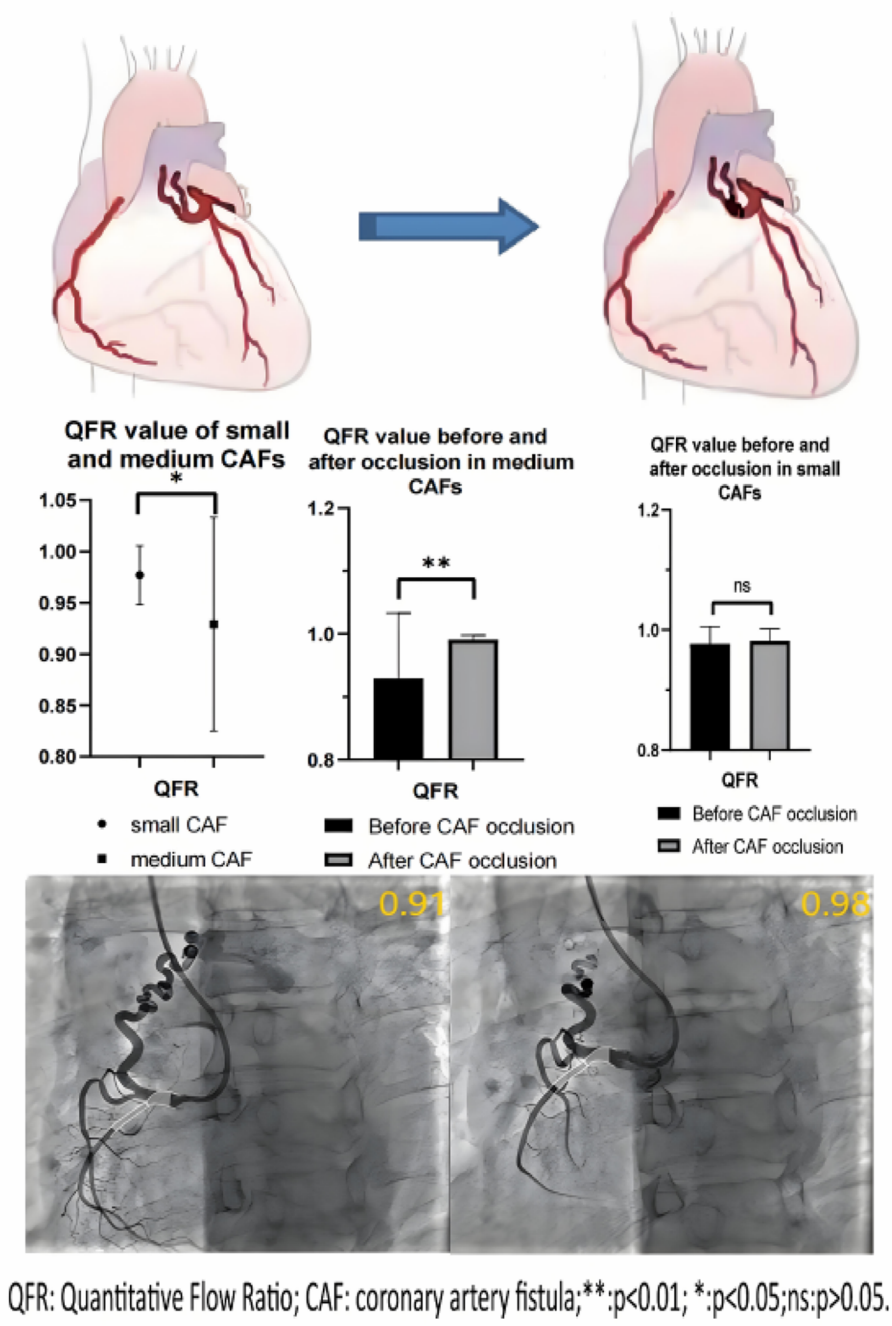
Central illustration.the QFRs of donor vessels with CAFs before and after occlusion.

None of the donor vessels associated with coronary artery fistulas exhibited significant atherosclerotic stenosis in enrolled patients. The mean QFR value in the donor vessels of CAFs was 0.96 ± 0.08. The mean QFR value of the donor vessels with medium CAFs was lower compared to those with small CAFs (0.93 ± 0.10 vs. 0.98 ± 0.03; *p* < 0.05) (Figure 1). The mean IMR values (2.66 ± 0.62 vs. 2.69 ± 0.59) in two groups of patients were no statistical differences (*p* > 0.05). Meanwhile, the changes in QFR were more significant in donor vessels with medium CAFs compared to those with small CAFs following CAFs occlusion (0.06 ± 0.10 vs. 0.005 ± 0.012; *p* = 0.01). Similarly, the QFR variation rate exhibited statistical difference (8.4% ± 15.9% vs. 0.5% ± 1.3%; *p* = 0.02). Regardless of whether the donor vessel was larger than CAFs, the changes in IMR variations and IMR variation rate were no statistical differences (*p* > 0.05) (Table 2).

**Table 2:** The influence of different CAFs sizes on the QFR of donor vessels.

	Small CAF (*n* = 26)	Medium CAF (*n* = 22)	*P*-value
QFR	0.98 ± 0.03	0.93 ± 0.10	0.03*
IMR	2.66 ± 0.62	2.69 ± 0.59	0.85
QFR variations	0.005 ± 0.012	0.06 ± 0.10	0.01*
QFR variation rate (%)	0.5 ± 1.3	8.4 ± 15.9	0.02*
IMR variations	0.08 ± 0.73	−0.02 ± 0.63	0.59
IMR variation rate (%)	7.62 ± 27.83	1.15 ± 27.23	0.42

QFR, quantitative flow ratio; IMR, index of microcirculatory resistance.

*P < 0.05.

**Figure 2:**
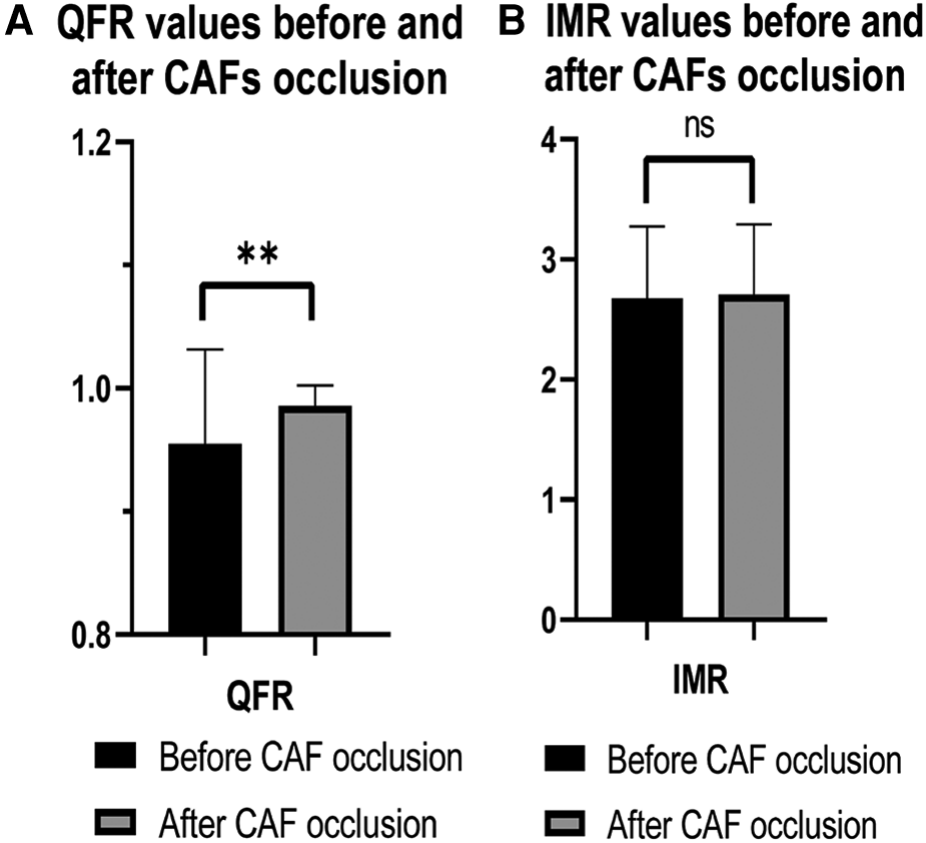
The QFR and IMR values changed before and after CAFs occlusion. (**A**) Following CAF occlusion, the mean QFR value of donor vessels was significantly improved compared to that observed before occlusion; (**B**) The mean IMR value remained unchanged after occlusion. CAF, coronary artery fistula; QFR, quantitative flow ratio; IMR, index of microcirculatory resistance; **: *p* < 0.01; ns: *p* > 0.05.

**Figure 3:**
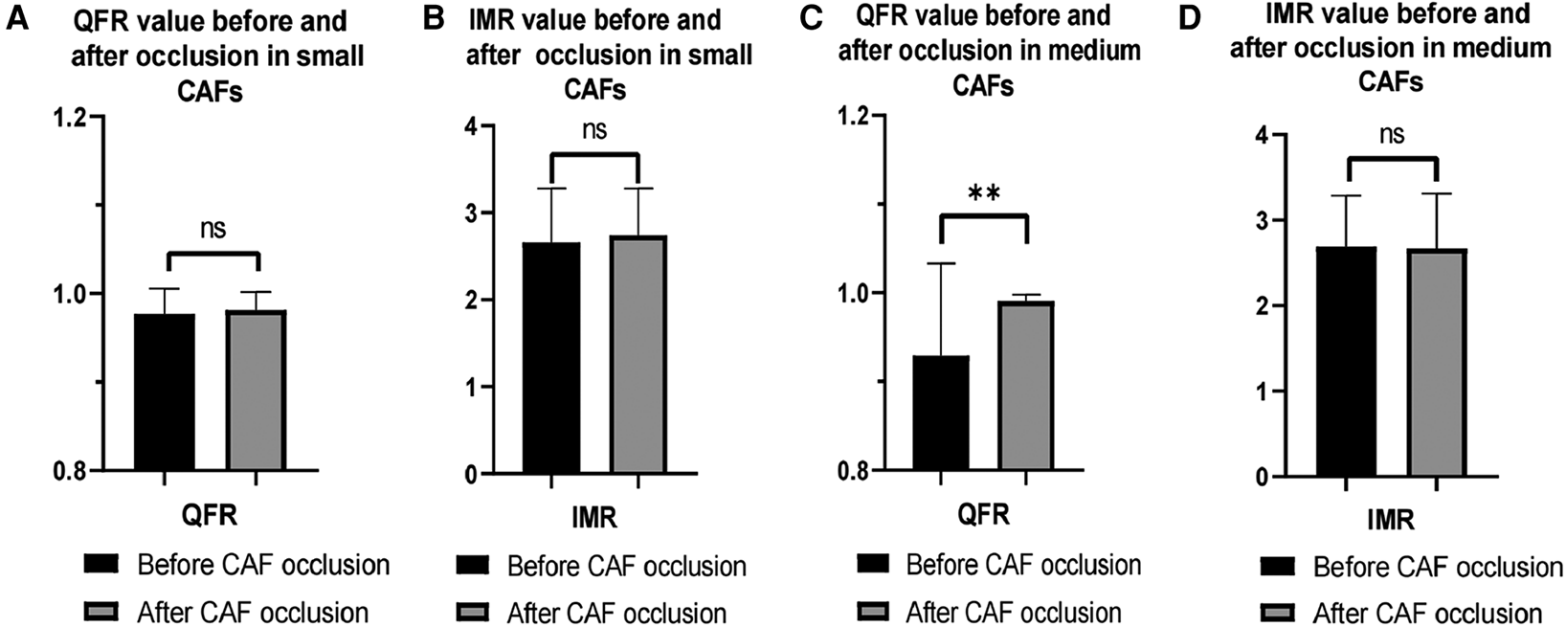
Changes in QFR and IMR values of patients with different CAF sizes before and after occlusion. (**A,B**) The mean QFR and IMR values were no statistical differences before and after CAF occlusion in donor vessels with small CAF; (**C**) The mean QFR value of donor vessels with medium CAF was significantly increased after CAF occlusion; (**D**) The mean IMR value was no significant difference in donor vessel with medium CAF before and after CAF occlusion. QFR, quantitative flow ratio; IMR, index of microcirculatory resistance; CAF, coronary artery fistula; **: *p* < 0.01; ns: *p* > 0.05.

**Figure 4:**
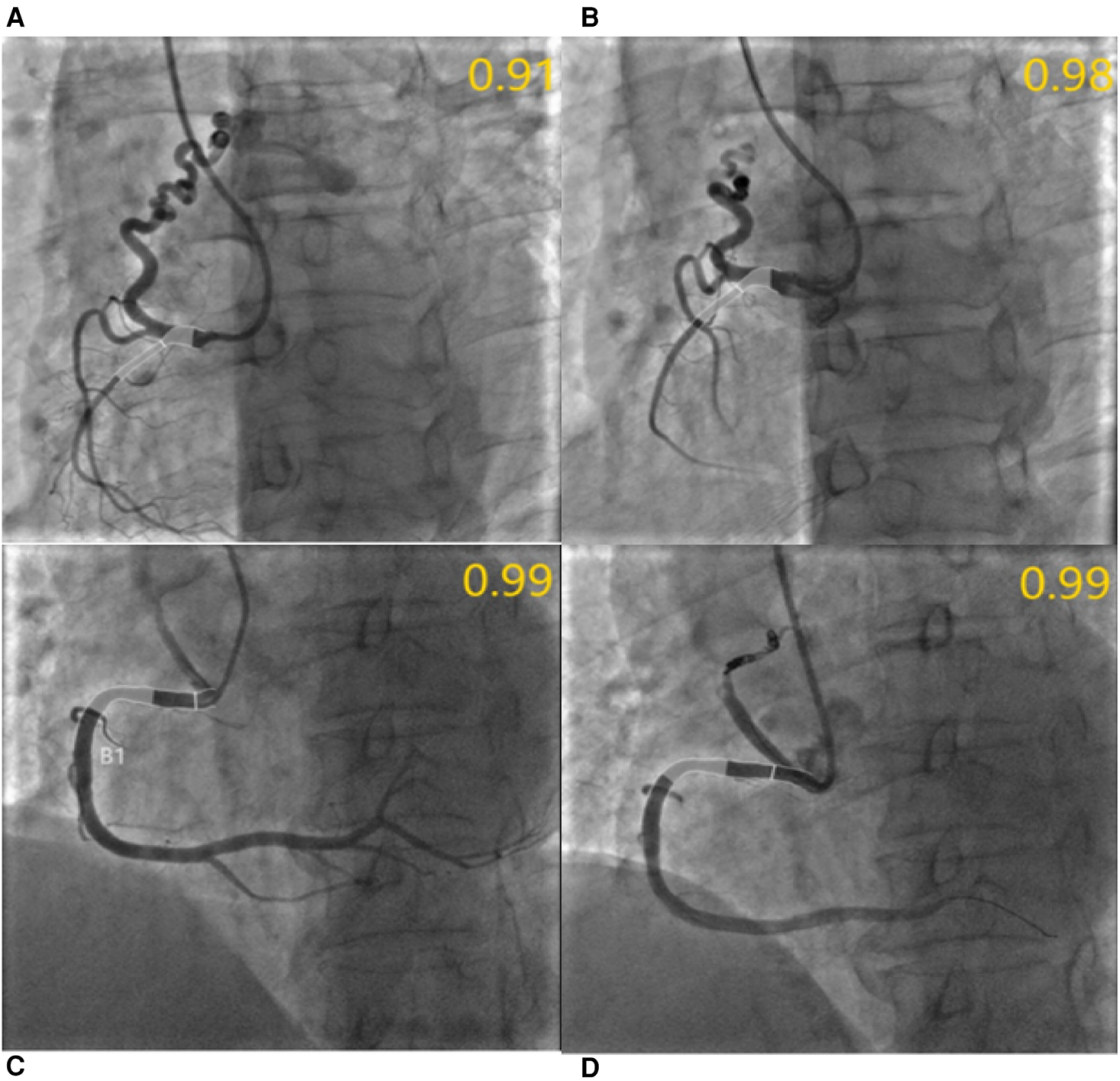
QFR assessment before and after CAFs occlusion. (**A**) Medium fistula with an ostial diameter of 4.0 mm originates from RCA with a diameter of 2.1 mm after the fistula. The QFR of RCA was 0.91 before occlusion; (**B**) The QFR of RCA was 0.98 after transcatheter closure using three coils; In another patient, the QFR of RCA with small fistulae was 0.99 before and after occlusion, respectively. (**C,D**) Meanwhile, the diameter of the RCA segment after fistulae was 4.7 mm and the diameter of the fistulae ostium was 3.8 mm.

Furthermore, following the occlusion of CAFs, the mean QFR value of donor vessels demonstrated a notable enhancement in comparison to the preceding state (0.99 ± 0.02 vs. 0.96 ± 0.08; *p* < 0.01). The IMR value remained unaltered following occlusion (*p* > 0.05) (Figure 2). Additionally, the mean QFR value of donor vessels with small CAFs exhibited no statistically significant distinction between the pre-and post-occlusion states (0.98 ± 0.03 vs. 0.98 ± 0.02; *p* > 0.05). The mean IMR value was not statistically different before and after CAFs occlusion in donor vessels with small CAFs (*p* > 0.05). However, the mean QFR value of donor vessels with medium CAFs exhibited a significant improvement following CAFs occlusion (0.99 ± 0.01 vs. 0.93 ± 0.10; *p* < 0.01). The IMR value remained statistically unchanged before and after CAFs occlusion in these donor vessels (2.69 ± 0.59 vs. 2.67 ± 0.65; *p* > 0.05) (Figure 3). Figure 4 presents the comparative QFR values of two examples with medium CAF (Figures 4A, B) and small CAF (Figures 4C, D) before and after CAFs occlusion.

Twenty-two (46%) of the 48 CAFs undergoing transcatheter closure exhibited no residual shunt after the procedure. However, there was no statistically significant difference in the mean QFR variation and QFR variation rate between donor vessels with CAFs that exhibited a residual shunt and those without residual shunt after occlusion, respectively (*p* > 0.05). Regardless of the presence or absence of a residual shunt, there was no statistically significant difference in the variation and variation rate of IMR (*p* > 0.05) (Table 3).

**Table 3:** QFR changes between residual shunt and not.

	No residual shunt after occlusion (*n* = 22)	Residual shunt after occlusion (*n* = 26)	*P*-value
QFR variations	0.05 ± 0.10	0.01 ± 0.02	0.06
QFR variation rate (%)	7.38 ± 16.21	1.32 ± 2.51	0.07
IMR variations	0.22 ± 0.76	−0.13 ± 0.57	0.08
IMR variation rate (%)	13.73 ± 32.77	13.02 ± 19.53	0.13

QFR, quantitative flow ratio; IMR, index of microcirculatory resistance.

## Discussion

To the best of our knowledge, this is the first study to evaluate the functional impact on the donor vessel following the closure of coronary fistulas using QFR analysis. The findings of this study are as follows: (1) In comparison to small CAFs, medium CAFs exert a more pronounced functional influence on the blood flow of the donor vessel. (2) The occlusion of CAFs enhances the functionality of donor vessels in cases where the CAFs are of a medium grade. Nevertheless, the improvement is not statistically significant in the case of small CAFs. (3) The presence of a small residual shunt following CAFs occlusion does not appear to affect the function of donor blood flow.

The steal phenomenon in CAFs is functionally significant, yet challenging to diagnose due to the limitations of routine imaging in assessing the degree of ischemia in these cases. The steal phenomenon occurs because the fistula provides a path of lower resistance for the blood flow, causing it to bypass the myocardial tissue that needs it the most during increased demand, such as during exercise or pharmacological stress. When coronary artery disease (CAD) coexists with a coronary artery fistula, the steal phenomenon can be more pronounced. In the presence of a significant stenosis, the myocardial perfusion is already compromised. The addition of a fistula exacerbates this condition by further redirecting blood flow away from the myocardium. During stress, this diversion becomes more critical, leading to myocardial ischemia. The hemodynamic severity of CAFs shunts can be evaluated by quantifying the proportion of blood flow redirected from the fistula to the receiving chamber (9).

Fractional flow reserve (FFR) has been widely adopted as the gold standard for assessing the functional severity of coronary artery disease (10, 11). Recently, the use of FFR to assess the functional improvement of the donor vessel after CAFs temporary or permanent occlusion has been reported (12–14). Mehdi et al. (15) reported that a clear improvement in the FFR value at 0.95 was observed after the closure of the fistula compared with the FFR value at 0.80 before closure under maximal hyperemia. The recent study highlights QFR correlates better with FFR in detecting hemodynamically significant CAD in patients with prior CAD, outperforming traditional myocardial perfusion imaging (MPI) methods like SPECT, PET, and CMR (16). This suggests that FFR might be superior to MPI for evaluating the CAFs. This is due to FFR's ability to provide a direct measurement of the pressure gradient across a coronary lesion, offering a more precise assessment of the physiological impact of the fistula on coronary blood flow.

QFR is an innovative angiographic-based technique that employs modern software for three-dimensional vessel reconstruction, and flow model calculation. A substantial body of evidence from numerous studies has consistently demonstrated a robust correlation between QFR and FFR in the assessment of coronary artery function (17, 18). Previous studies have also shown a significant association between QFR values and the physiological function of coronary atherosclerotic stenosis (19, 20). Westra et al. (21) demonstrated that for every 0.10 increase in QFR, there was an increase in ^82^Rb PET stress myocardial blood flow by 0.08 ml/g/min (95% CI: 0.02–0.14 ml/g/min) among patients with intermediate coronary artery stenosis. In our study, the donor coronary arteries with CAFs demonstrated no notable stenosis, indicating that the QFR value is associated with the physiological function of the donor vessels following CAFs drainage in these patients.

In accordance with the current guidelines, a small or medium fistula should be closed in patients presenting symptoms of myocardial ischemia, arrhythmias, ventricular dilation or dysfunction, or endarteritis (22). Although the majority of CAFs are asymptomatic, it has even been reported that small CAFs can spontaneously close over time in children (23). Recent evidence indicates that in individuals aged under 20 years, only one-fifth of the fistulas are symptomatic, while more than two-thirds of the fistulas are symptomatic after the age of 60 years (24). In our study, the enrolled patients ranged in age from 39 to 84 years, with over 75% of them being older than 60 and presenting symptoms of myocardial ischemia, heart failure, or arrhythmia.

The utilization of QFR analysis in this study confirmed that small CAFs exert no influence on donor blood flow, revealing no noteworthy improvement following occlusion. Conversely, the medium CAFs occlusion demonstrated the capacity to improve donor blood flow function, aligning with the prevalent opinion (7). The notable enhancement in QFR values, particularly in medium-sized CAFs, substantiates the hemodynamic advantages of CAF occlusion. This improvement indicates that the closure of CAFs can effectively enhance coronary blood flow, potentially alleviating myocardial ischemia. However, the lack of significant changes in IMR values indicates that microvascular resistance remains stable post-occlusion. This finding suggests that the primary benefit of CAF closure is likely related to improved macrovascular flow dynamics rather than changes in microvascular function. The differentiation between medium and small CAFs in terms of QFR improvement can aid in risk stratification. Medium-sized CAFs may require more aggressive intervention to achieve optimal outcomes. Notably, this conclusion has been validated through QFR for the first time.

Currently, assessing the immediate effects of CAFs closure is primarily based on symptom relief and imaging techniques to confirm the patency of the closure site and residual shunting. The treatment approach for residual shunts during follow-up after CAFs occlusion mainly involves re-occlusion (25, 26). However, research examining the functional impact of residual shunts following CAFs occlusion on the donor blood flow remains limited. Our research reveals that minimal residual shunts following the CAFs occlusion exert no significant influence on the donor blood flow. There is no discernible difference in QFR variation between residual shunts and no residual shunts. The absence of significant differences in QFR and IMR variations between CAFs with and without residual shunt implies that incomplete occlusion can provide substantial hemodynamic benefits. This may be attributed to the fact that the residual shunts immediately following occlusion tend to close spontaneously during the follow-up period (26). This finding is clinically relevant as it highlights the potential utility of CAFs occlusion in patients where complete closure is not achievable.

## Conclusion

This study initially evaluates the physiological impact on blood flow within the donor vessel following CAFs closure through QFR analysis. The presence of medium CAFs has a significant impact on the blood flow of the donor vessel, as compared to small CAFs, and may benefit from occlusion. A small residual shunt has no significant impact on the effectiveness of CAFs occlusion in enhancing donor blood flow.

## Limitations

This study has several limitations. The retrospective study focuses only on the physiological impact of donor vessels immediately following CAFs occlusion but lacks clinical follow-up data to validate the long-term symptomatic and functional improvements. Furthermore, the enrolled cases exclusively consisted of small and medium-sized CAFs, while the majority of large CAFs underwent surgical ligation, resulting in unavailable QFR data post-procedure.

## Data Availability

The raw data supporting the conclusions of this article will be made available by the authors, without undue reservation.
